# Surgical Treatment of Tethered Cord Syndrome by Release of Filum Terminalis: A Review

**DOI:** 10.3390/children13040534

**Published:** 2026-04-12

**Authors:** Marios Lampros, Flavio Giordano, Panagiota Zagorianakou, George A. Alexiou, Spyridon Voulgaris

**Affiliations:** 1 Department of Neurosurgery, School of Medicine, University Hospital of Ioannina, 45500 Ioannina, Greece; marioslampros@gmail.com (M.L.); panzagorian@gmail.com (P.Z.); svoulgar@uoi.gr (S.V.); 2 Medical School, University of Ioannina, 45500 Ioannina, Greece; 3 Functional Neurosurgery and Epilepsy Neurosurgery and Pediatric Neurosurgery Unit Meyer Children’s Hospital IRCCS Florence, 50139 Firenze, Italy; flavio.giordano@unifi.it; 4 Department NEUROFARBA, University of Florence, 50019 Firenze, Italy

**Keywords:** tethered cord syndrome, filum terminale, detethering

## Abstract

Tethered cord syndrome (TCS) is a disease caused by pathological fixation of the spinal cord, most commonly due to a thickened filum terminale, postoperative adhesions, or congenital dysraphism. Progressive neurological, urological, and orthopedic manifestations result from chronic cord traction and impaired vascular supply. Surgical detethering remains the standard treatment, with the classic intradural sectioning of the filum terminale being the most widely used technique. Recent developments, however, include minimally invasive tubular and endoscopic approaches, spinal column shortening procedures for recurrent or complex cases, and extradural detethering strategies. Each technique aims to reduce cord tension while minimizing postoperative complications, particularly cerebrospinal fluid leakage and retethering. This review summarizes the anatomical background, pathophysiology, and operative strategies for TCS, highlighting current evidence, technical nuances, and limitations of emerging minimally invasive and alternative approaches.

## 1. Introduction

**Figure 1:**
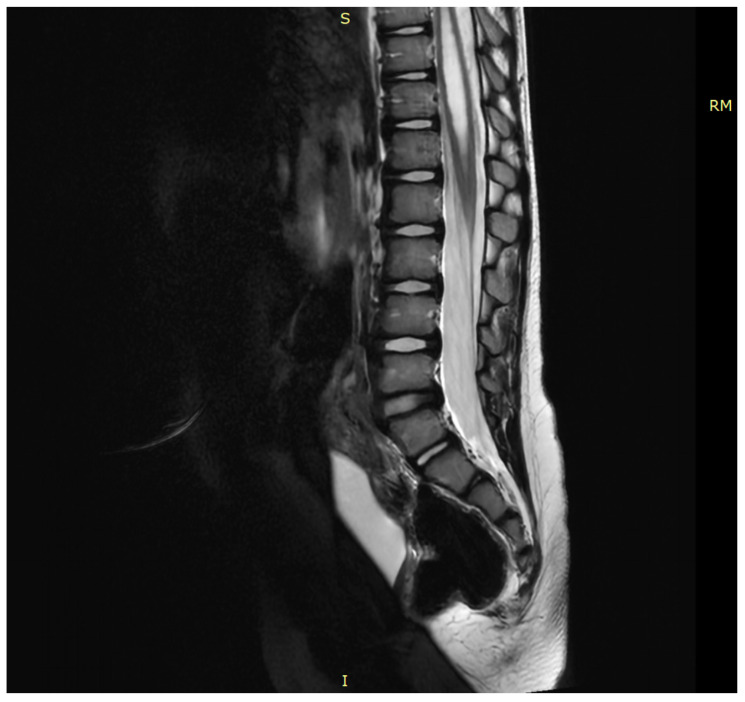
Low-lying conus medullaris in a sagittal T2W-MR scan associated with tethered cord syndrome (TCS) caused by tight filum terminalis and leading to syringomyelia.

Tethered cord syndrome (TCS) is a neurological disorder characterized by caudal displacement of the spinal cord and an abnormally low-lying conus medullaris (Figure 1) [1]. Typically, TCS is the result of anchoring of the spinal cord and dura to inelastic connective tissue, with anchoring in a pathologically short and thickened filum terminalis in a patient previously operated on for myelomeningocele (MM) being the primary cause [2]. Approximately 70% of the patients with MM will have radiographic evidence of TCS and 20–30% of them will become symptomatic. Other disorders associated with pathological tethering include closed dysraphism (lipomyelomeningocele, split cord malformation (SCM)) and scar formation following spinal operations or trauma [3]. While most cases of TCS present in childhood, adult cases of TCS have been rarely reported [2,3]. Symptoms of TCS are associated with spinal cord stretch and usually occur during the growth-spurt and include lower limb spasticity, foot deformities, bladder dysfunction, and lower limb and perianal pain [4,5,6,7].

After confirmation of TCS with magnetic resonance imaging (MRI), surgical detethering is usually recommended for symptomatic patients [7,8,9,10,11]. However, cases in which the patient has symptoms highly suggestive of TCS without imaging evidence have been described in the literature, and this clinical entity is described as occult TCS [8]. The typical surgical technique of detethering was first described by Garceau in 1953 [9,12]. However, based on retrospective cohort studies, the procedure has been linked with a significant rate of cerebrospinal fluid (CSF) leak (5–15%) and symptom recurrence from postoperative scar formation [10,11]. Currently, less invasive techniques with the use of an endoscope or via an extradural approach have been described [13,14,15].

While several reviews on TCS and the filum terminale sectioning have been published in recent years, only a few have focused specifically on the technical aspects of surgical detethering. The present review aims to complement the existing literature by offering an up-to-date, technique-centered synthesis of all major surgical approaches, with particular emphasis on practical anatomical considerations, procedural nuances, and the comparative limitations of each method.

## 2. Literature Search Strategy

A literature search was conducted across three major electronic databases: PubMed (MEDLINE), Scopus, and EMBASE. The search was performed without restriction on publication date, with the last search update completed in December 2025. The following search terms were used in various combinations: “tethered cord syndrome”, “tethered spinal cord”, “filum terminale”, “filum terminalis”, “filum section”, “filum sectioning”, “detethering”, “spinal cord detethering”, “conus medullaris”, “spinal dysraphism”, “myelomeningocele”, “occult tethered cord”, “minimally invasive spine surgery”, “endoscopic spine surgery”, “spinal cord untethering”, “vertebral column shortening”, “extradural detethering”, and “lumbosacral surgery”. Articles were included if they reported on the surgical management, anatomical considerations, pathophysiology, outcomes, or complications of tethered cord syndrome or filum terminale sectioning, in either pediatric or adult populations. No language restriction was applied; however, only articles available in full text or with sufficiently detailed abstracts were considered. Case reports, case series, retrospective and prospective cohort studies, and review articles were all eligible for inclusion. Studies entirely unrelated to surgical technique or clinical outcomes of TCS were excluded. Reference lists of retrieved articles were also manually screened to identify additional relevant publications not captured by the database search.

## 3. Anatomical Considerations

The filum terminalis is the caudal extension of the conus medullaris which originates from the pia mater and is directed caudally, penetrating the dural cul-de-sac and continuing extradurally as the coccygeal ligament which anchors the spinal cord to the periosteum of coccyx [1,16,17]. The intradural part is also called the filum terminale internum (FT-I), while the extradural part or the coccygeal ligament is called the filum terminale externum (FT-E).

The whole length of the filum terminale is approximately 20 cm long, with FT-I being the longest, approximately 75% of the total length or about 15 cm, while the FT-E measures about 5 cm [18,19]. While it originates from the pia mater and is usually described as a “fibrous-band”, its histopathological composition is more complex, especially in the intradural portion, as first described by the cadaveric study of Harmeier et al. in 1933 [18]. Specifically, several cell populations have been described in the FT-I including glial cells such as astrocytic, ependymal cells, oligodendrocytes, arachnoid cells that form subarachnoid cells, and the presence of corpora amylacea, small hyaline masses formed from polyglucosan related with degeneration of connective tissue, has also been described [9,19]. These cells are probably remnants of the secondary neurulation stage or stage of retrogressive differentiation [20]. Interestingly, the ependymal cells located in the conus medullaris and the filum terminale have been found to be the cells of origin of myxopapillary ependymomas [21]. However, the main population of cells is from mesenchymal connective tissue cells that participate in the synthesis of dense type I collagen and extracellular matrix which forms the backbone of the filum terminale [22].

**Figure 2:**
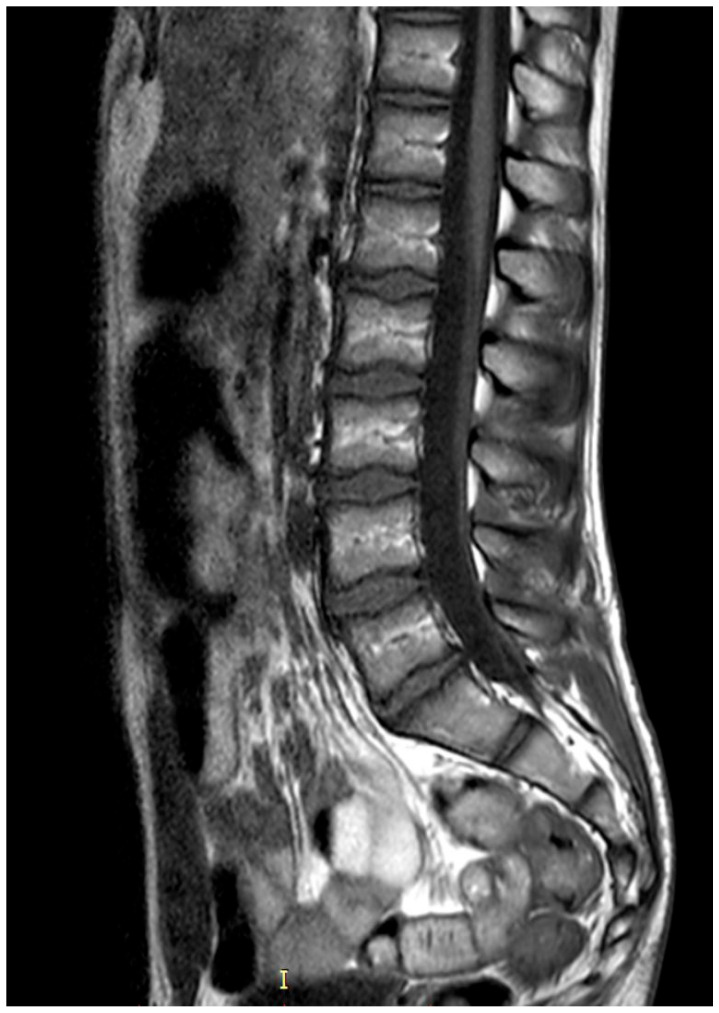
Young patient with non-specific lower limbs symptoms. The conus medullaris is at L1/L2 level without clear diagnosis of TCS. Spinal cord release is not indicated.

At birth, the conus medullaris is usually located at the level of L3, but afterwards, the vertebral column and dura grow faster and disproportionately relative to the spinal cord in a rostral direction, and as a result the conus medullaris is found at the level of L1 or L2 in adulthood [23]. This disproportionate growth occurs even in fetal life and is accelerated during two distinct phases: during infancy (early growth spurt) and during the childhood growth spurt (around 5–6 years) [24]. In cases of TCS, the “ascending” of the conus medullaris is limited due to the anchoring of the spinal cord to the surrounding vertebrae and musculoskeletal tissues resulting in a lower-lying spinal cord below the level of L2 [25]. These pathological alterations result in the stretching of the filum terminale and conus medullaris and in their related blood vessels, causing local ischemia. This ischemia from vessel stretching has been linked with the manifestations of TCS symptoms [26]. In some cases, the conus medullaris may be at a normal level, but the stretching of vessels may be present and cause symptoms. The latter is the primary pathophysiological mechanism of occult TCS (OTCS) [27]. The existence of OTCS, however, remains one of the most debated topics in spinal neurosurgery. Symptomatic improvement following filum sectioning in the absence of imaging abnormality has been reported, constituting indirect evidence of pathological tethering [8,27], and histological studies of the filum in OTCS patients have revealed structural abnormalities not detectable on standard MRI, suggesting that conventional imaging may underestimate the degree of pathological change [22]. On the other hand, the diagnosis relies on nonspecific symptoms—including back pain, lower limb weakness, and bladder dysfunction—that overlap considerably with other conditions, and the reported surgical benefit has been questioned given the lack of objective outcome measures and controlled data [28]. The absence of validated diagnostic criteria further complicates patient selection, and no consensus currently exists on the indications for surgery in this population (Figure 2).

**Figure 3:**
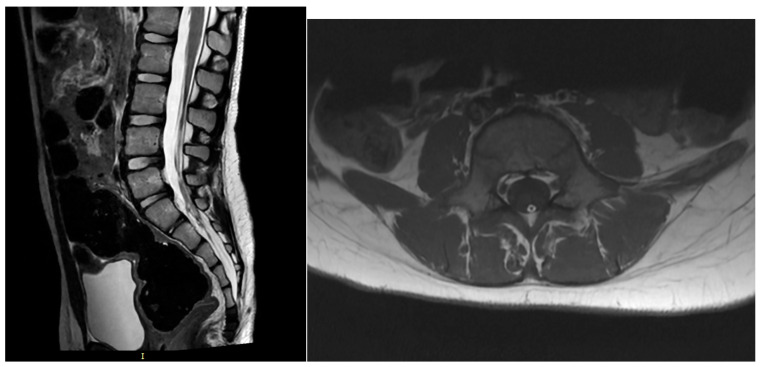
Tight and fatty filum terminalis causing a typical tethered spinal cord syndrome on sagittal T2-MR scan on the left and axial T1W-MR scan on the right.

**Figure 4:**
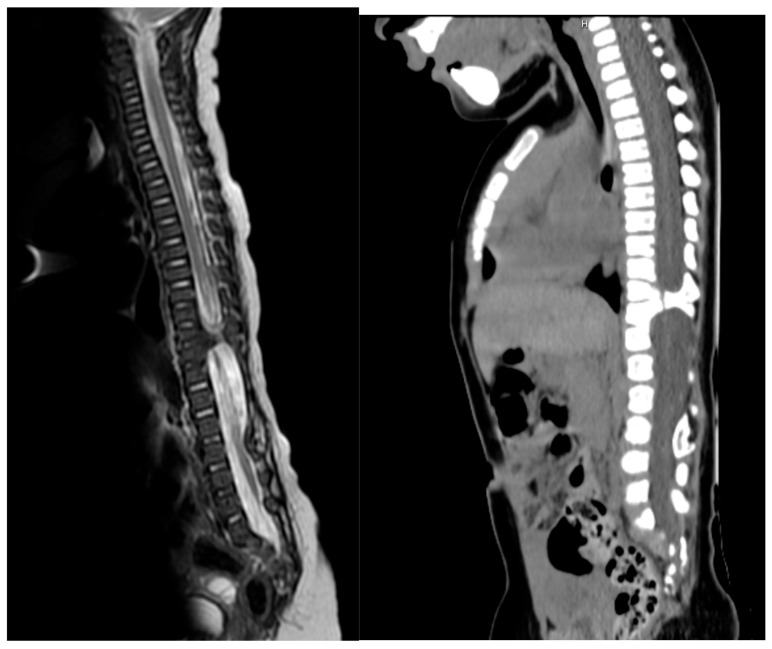
Split cord malformation (SCM) type I due to vertebral bony spur, as seen on sagittal T2W-MR on the left and on sagittal CT-scan on the right side.

**Figure 5:**
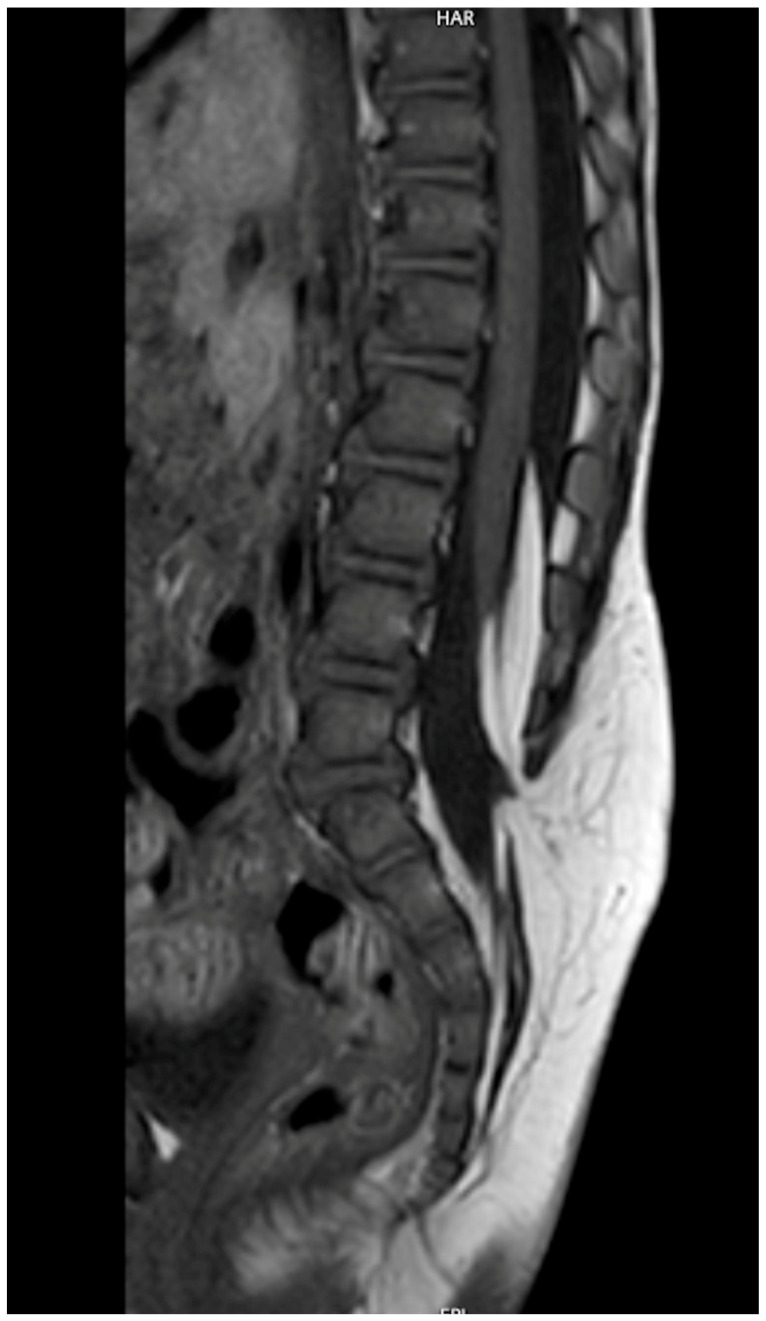
Lipomyelocele—also known as caudal or dorsal spinal lipoma in sagittal T1W-RM.

**Figure 6:**
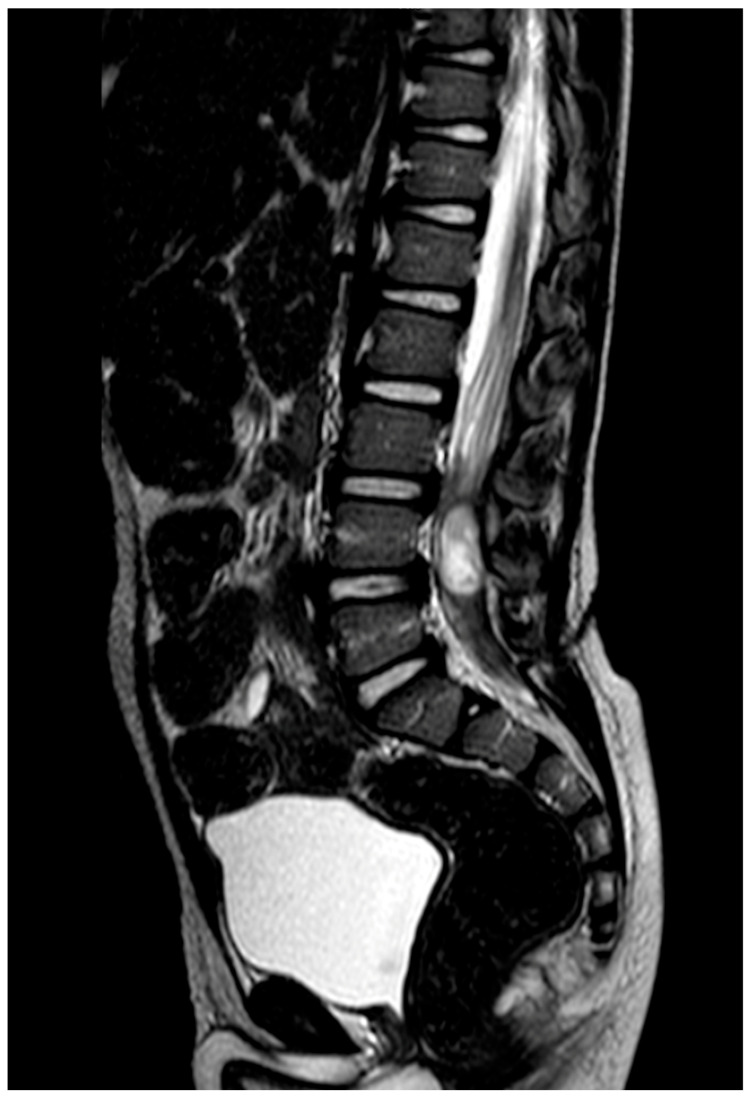
Sagittal T2W-MR showing a dermal sinus tract causing spinal tethering associated with epidermoid cyst.

TCS can be classified as congenital (primary) or acquired (secondary) [29]. Primary TCS is traditionally characterized by a congenitally thick, fatty and short filum terminale, which may occur in both syndromic, usually in spinal dysraphism, and non-syndromic contexts (Figure 3) [1]. Secondary TCS results from an anchoring of the filum terminale to inelastic tissue, most commonly due to postoperative or traumatic scarring, or less frequently, the presence of a spinal neoplasm. However, that classification is not widely utilized since in most cases of patients with MM, the most common syndromic disorder associated with TCS, the tethering of the spinal cord is the result of the formation of postoperative adhesions following MM repair rather from the dysraphism itself, and the FT is not thickened and short at birth [30]. Finally, in patients with other types of dysraphism, the mechanism of tethering varies by entity. In split cord malformation (SCM, Figure 4), the spinal cord is tethered by a rigid bony or cartilaginous septum, while in spinal lipomas (Figure 5) and dermal sinus tracts (Figure 6), anchoring results from inelastic fibro-adipose tissue or lipomas [31].

## 4. Classic Technique

The typical procedure is performed via an intradural approach, aiming to remove the adhesions from the spinal cord and nerve roots, followed by sectioning of the FT-I [32]. The typical indications include progressive neurological deficits, bladder dysfunction, limb spasticity and deformities, and intense pain. A recent MRI of the lumbar spine is required preoperatively to confirm a low-lying cauda equina, its anatomical position relative to the vertebrae, a thickened and shortened filum terminale, and to reveal associated congenital abnormalities such as lipomyelomeningocele, lipomas, and dermal sinuses [33]. A urodynamic study is also performed preoperatively to assess the presence and extent of sphincter dysfunction, which can then be used as a reference for postoperative urodynamic follow-up assessment [34]. The surgery is performed under general anesthesia and intraoperative neurophysiological monitoring is recommended to confirm safe resection and to avoid the damage of nerve roots, especially motor roots [35].

The patient is placed in the prone position with the use of supporting rolls bilaterally, and a midline skin incision in the lower lumbar region is made depending on the level of tethering and FT-I, usually between L4 and S1, followed by paraspinal detachment of the paraspinal muscles. Paramedian incisions have also been described in the literature. Laminectomy or laminotomy of the L4 and L5 vertebrae is typically performed with the use of Kerrison rongeurs or a high-speed drill, followed by the removal of the ligamentum flavum [35,36]. In cases where a congenital defect is present, the laminectomy should carefully start from healthy tissue to avoid any neural injury. Similarly, after completion of the laminectomy, the dura should be adequately exposed with clear, healthy margins. The dura is initially opened in the midline with the use of a No. 11 scalpel blade and the incision is extended with the use of a Taylor dural scissor [37]. The dural edges are retracted bilaterally and tacked with the use of sutures. Then, a microscope is placed in the operating field and arachnoid dissection is performed aiming to identify the FT-I and to remove pathological tissues such as lipomas or scars responsible for the tethering [38,39].

Identification and discrimination of the FT-I from the adjacent nerve roots is a critical point in the operation of TCS. The FT-I is typically located in the midline, dorsally, and is extended caudally from the conus medullaris [40]. It is typically pale, glistering, and has an associated filar artery and vein, with the vein placed posteriorly from the artery [40,41]. Interestingly, arteriovenous fistulas have been described between the filar artery and vein [42]. These anatomical features along with the experience of the surgeon may help in the identification of the FT-I. Intraoperative neurophysiological monitoring can also be used to discriminate FT-I from nerve roots [43]. Specifically, stimulation of a motor root may elicit electromyographical (EMG) responses at low thresholds, while the filum does not elicit a response, even in higher thresholds [44]. Then, the FT-I is carefully cauterized approximately 3–5 mm from its attachment to the conus medullaris. Finally, after the detethering of the spinal cord, resection of pathological tissues, and repair of associated congenital abnormalities, the dura is closed in a watertight fashion and sealed to prevent postoperative CSF leakage [44,45]. In cases involving laminotomy, fixation may be performed to stabilize the lamina [46]. Hemostasis is then achieved, followed by layered closure of the paraspinal muscles and skin [47].

## 5. Minimally Invasive Techniques

Apart from the traditional techniques for TCS, several less invasive procedures have been described that typically include the use of tubular or cylindrical retractors to access the operating site and theoretically reduce soft tissue injury and minimize the risk of postoperative complications [48,49]. In 2007, Tredway et al. [48] first described the use of tubular retractors for spinal cord detethering in three patients with TCS. As in the typical procedure, the patient was placed prone, and muscle splitting was performed at the L4–L5 interspace with the use of dilators. A 22 m cylindrical retractor was then placed, and a midline laminotomy of the inferior part of the L4 level and of L5 was performed followed by a small, about 1 cm, incision and arachnoid dissection. Then, the typical procedure for filum terminale identification, ligation, and cut was followed with the use of intraoperative neurophysiological monitoring. In another small case series study, Potts et al. [49] also utilized expandable retractors to perform minimally invasive detethering in three adult patients with tethered cord syndrome. Compared with the previous study conducted in children, this adult study performed a laminotomy at the L2–L3 intervertebral space and compared several surgical parameters with those of the traditional technique, which was also applied in three adult patients with tethered cord syndrome (TCS). The authors reported that the mean estimated intraoperative blood loss and the mean length of hospital stay were similar between the two groups. However, the incision length in the mini-open group was reduced by half, although one patient in this group required revision surgery for pseudo meningocele. The efficacy of the minimally invasive approach was also supported by a case series with 11 patients [50], which reported no major complications at the follow-up. More recently, in 2017, Telfeian et al., in a case report, described a novel minimally invasive endoscopic approach in an adult patient with tethered cord syndrome (TCS) and lipomyelomeningocele. The procedure began with a 2 cm midline incision at the L4–L5 intervertebral level, through which a 7 mm endoscopic tubular retractor was placed, followed by laminotomy of L5 using an endoscopic drill. The tubular retractor was then removed and replaced with an expandable minimally invasive retractor. Through this, a 7 mm vertical dural incision was performed, allowing identification of the filum terminale. The filum was grasped endoscopically, coagulated with bipolar electrocautery, and subsequently transected with micro-scissors [51]. Currently, and due to the low incidence of TCS, there are no large cohorts to compare open vs. minimally invasive approaches. Hence, it is unclear whether the minimally invasive approach reduces the risk of postoperative complications.

## 6. Spinal Column Shortening

Despite the development of novel and minimally invasive techniques for the treatment of tethered cord syndrome (TCS), their efficacy remains variable, with a large retrospective study with 342 patients reporting rates of retethering ranging from 5% to 50% [52]. Higher rates are typically observed in patients with complex forms of TCS, particularly those associated with lipomyelomeningocele or myelomeningocele. In such high-risk cases, or in instances of recurrent retethering complicated by issues such as cerebrospinal fluid (CSF) leakage and nerve root damage, an alternative surgical approach has been described. This method aims to reduce longitudinal tension on the spinal cord, operating on the principle of decreasing stress in a manner analogous to relieving strain on a tightly stretched rope. The latter technique is called spinal column shortening (SCS) and may include several variations in vertebral column subtraction (VCS) such as posterior vertebral column subtraction osteotomy (PVCSO) and homogenous spinal-shortening axial decompression (HSAD) [52,53,54]. The typical procedure of SCS for TCS was originally described by Kokobun et al. in a series of papers starting from 1995 [55] to more recent series of publications [56]. The L1 vertebra is typically selected as the site for osteotomy in spinal cord shortening procedures. The operation begins with removal of the lower half of the T12 pedicle and the upper one-third of the L1 pedicles, together with the T12–L1 facet joints. Pedicle screws are inserted into the T12 and L2 vertebrae to provide segmental stabilization of the spinal column. Subsequently, the upper three-quarters of the L1 pedicles are excised, along with the upper half of the L1 vertebral body, the posterior longitudinal ligament, and the T12–L1 intervertebral disc. The resulting gap is then gradually closed by applying compression through the pedicle screws and securing the construct with shorter connecting rods, thereby achieving controlled spinal column shortening and reduction in longitudinal cord tension. A schematic explanation of the procedures can be found in the original article [56]. PVCSO is an alternative procedure to reduce the height of the spinal cord in cases of recurrent TCS involving laminectomy from T11 to T2, pediculotomy of T12, removal of T12 proximal ribs, discectomy of T11–T12 intervertebral disc and partial osteotomy of the upper segment of T12 with the use of a high-speed drill. This procedure results in a total reduction of approximately 15–25 mm of spinal column height [57]. HSAD is an alternative procedure in which a midline approach is performed to expose the spinous processes, laminae, and facet joints from L1 to S1, and bilateral pedicle screws are placed at each level. At L1–L2, the interspinous ligament is excised, and the inferior articular process of L1 along with the superior portion of the L2 lamina are resected to enlarge the intervertebral foramen. The L1–L2 disc is removed and replaced with autologous bone graft, and the same sequence of decompression and fusion is carried out from L2–3 through L5–S1. Controlled compression is applied at each segment to restore alignment, reduce interspinous gaps, and decompress the dural sac and nerve roots [58]. The efficacy and safety of SCS was recently evaluated by a systematic review and meta-analysis of 15 studies that included 191 patients with TCS. The average age of patients at the time of surgery was 28 years, indicating that it is used primarily on non-pediatric cases with recurrent TCS. Approximately a 60–80% improved rate was reported for pain and weakness. However, improvement of bladder and bowel dysfunction was reported in less than half of the patients (47.4%), and postoperative complications were reported in four patients [59]. However, in another older systematic review and meta-analysis that included six studies with 57 patients the authors reported higher rates of improvement for urinary and bowel dysfunction, approximately 80–100%, with the authors concluding that the technique is generally safe and effective [60].

## 7. Extradural Detethering

Extradural detethering has been proposed as a theoretically less invasive alternative to conventional intradural sectioning of the filum terminale. In a small case series, Basilotta Marquez et al. described three cases of myelomeningocele-associated tethered cord syndrome in which scar tissue adherent to the dura was carefully dissected, and a Spongostan spacer was interposed between the dura and paraspinal muscles to prevent retethering, secured in place with lateral sutures. All three patients demonstrated postoperative improvement in symptoms, supporting the potential clinical utility of this technique [61]. Similarly, in a single case report, Veronesi et al. reported an adult case of TCS with the conus medullaris terminating at the S2 level, where the patient presented with severe dysesthesias and pain in the lower limbs. Management was achieved through an extradural transhiatal approach, in which the superficial dorsal sacrococcygeal ligament was removed to access the sacral canal. Within the canal, the filum terminale externum (FTE) was identified, isolated, and sectioned. The procedure lasted approximately 20 min, and at both 3- and 6-month follow-up, the patient demonstrated marked symptomatic improvement [62].

However, anatomic and cadaveric studies have questioned the mechanical efficacy of extradural detethering. Specifically, Patel et al., in a cadaveric biomechanical study, observed that tension applied to the FTE in cadaveric specimens produced only minimal movement of the FTI and no displacement of the conus medullaris, suggesting that isolated FTE transection may have a limited detethering effect [41]. Similarly, De Vloo et al., in a cadaveric anatomical and histological study evaluating the elastic properties of the filum, found that the FTI is highly elastic and primarily functions to absorb traction forces on the spinal cord. These findings support the clinical observation that, while extradural approaches may lead to subjective symptomatic improvement, their effectiveness is likely less related to direct mechanical detethering and more to the release of local adhesions and reduction in scar-related traction in small retrospective series regarding patients who have previously undergone spinal surgery [40]. Overall, the extradural detethering of the spinal cord remains a theoretical solution since it lacks any anatomical evidence, and to date no prospective double blind, randomized studies have been reported in the literature.

## 8. A Comparative Synthesis of Surgical Approaches

The surgical management of tethered cord syndrome has evolved considerably over the past two decades, expanding from a single dominant technique to a diverse armamentarium of approaches that differ fundamentally in their mechanism of action, invasiveness, and the clinical contexts in which they are best applied [1,7]. Taken together, the evidence reviewed here allows for several meaningful observations. A summary of the advantages and disadvantages of the current surgical approaches is presented in Table 1.

Classic intradural filum terminale sectioning remains the cornerstone of TCS surgery. Its long track record, anatomical directness, and well-characterized outcomes make it the reference standard against which all other techniques are measured [35,37]. The procedure is safe and effective in the primary setting, with neurological stabilization or improvement reported in the majority of patients [10,35]. Its principal limitations, CSF leakage in 5–15% of cases and symptomatic retethering driven by postoperative scar formation [3,10], have motivated the development of alternative approaches, yet none has demonstrated superiority in large, prospective comparative studies. Minimally invasive techniques, whether tubular or endoscopic, apply the same surgical principle as the classic approach but aim to reduce approach-related morbidity [48,49]. The available evidence, though limited by small series and the rarity of the condition, suggests comparable efficacy with reduced incision length [49,50]. However, the reduction in major complications such as CSF leak and retethering has not been convincingly demonstrated, and larger comparative studies are needed before minimally invasive approaches can be recommended as the new standard [50,51]. Spinal column shortening represents a conceptually distinct innovation, addressing cord tension indirectly rather than through direct cord manipulation [53,56]. By reducing the longitudinal height of the vertebral column, SCS bypasses the scarred surgical field that makes repeat intradural dissection hazardous [54,57]. Meta-analytic data support its efficacy for pain and motor symptoms, though improvements in bladder and bowel dysfunction remain less consistent [59,60]. Given its technical complexity and the profile of complications associated with major spinal osteotomy, SCS should be considered a second-line or salvage strategy in carefully selected patients with recurrent or complex TCS [54,59]. Extradural detethering occupies a unique and still unsettled position in this landscape [61,62]. While reported clinical outcomes in small series are encouraging, cadaveric studies have raised fundamental questions about the mechanical basis of the technique, suggesting that FTE transection produces negligible displacement of the conus medullaris [41]. The subjective improvement observed in patients may therefore reflect relief of local adhesion-related traction rather than true spinal cord detethering [40,41]. Until prospective, controlled data are available, extradural approaches should be regarded as investigational. In summary, the selection of the surgical technique should be driven by the underlying etiology, the presence or absence of prior surgery, the complexity of associated dysraphism, and the individual patient’s clinical trajectory [1,2,29]. Classic intradural filum sectioning remains the first-line choice in primary TCS [35,37], while minimally invasive refinements offer a reasonable alternative in straightforward cases [48,50]. SCS and extradural approaches address specific and distinct clinical niches [54,61,62], and their roles will likely become better defined as the evidence base matures.

## 9. Conclusions

Classic intradural filum terminale sectioning remains the standard of care for primary TCS, supported by decades of clinical experience and an acceptable safety profile. Alternative techniques, minimally invasive approaches, spinal column shortening, and extradural detethering, each address specific clinical scenarios where conventional surgery is technically challenging, carries excessive risk, or has previously failed. Despite the expanding range of available surgical options, robust comparative data remain scarce, and critical questions persist regarding the optimal management of recurrent TCS, the long-term durability of minimally invasive approaches, and the true mechanical efficacy of extradural detethering. Future prospective, multicenter studies with standardized outcome measures are needed to better define the role of each technique and guide individualized surgical decision-making.

## Figures and Tables

**Table 1:** Comparative overview of surgical approaches for tethered cord syndrome: indications, advantages, limitations, and level of evidence.

Technique	Primary Indication	Advantages	Main Limitation	Level of Evidence
Classic intradural filum sectioning	Primary TCS; thickened filum terminale [1,35]	Gold standard; direct cord release; well-established safety profile [37]	CSF leak (5–15%); symptomatic retethering from scar formation [3,10]	Moderate (large retrospective series) [35,37]
Minimally invasive (tubular/endoscopic)	Primary TCS in selected patients; adult cases [48,49]	Reduced incision length; less soft tissue disruption [49]	No proven reduction in CSF leak or retethering; limited comparative data [50,51]	Low (small series only) [48,49,50,51]
Spinal column shortening	Complex or recurrent TCS; retethering after MM or lipomyelomeningocele repair [54,56]	Avoids scarred intradural field; novel mechanism of tension reduction [53,57]	Technically demanding; major osteotomy risk; inconsistent improvement in bladder/bowel function [59,60]	Low–moderate (meta-analyses of small series) [59,60]
Extradural detethering	Recurrent TCS; extradural scar-related tethering [61,62]	Minimal invasiveness; avoids intradural dissection [62]	Lack of anatomical evidence for true cord detethering; no prospective data [40,41]	Very low (case reports and small series) [61,62]

Abbreviations: TCS: tethered cord syndrome; CSF: cerebrospinal fluid; MM: myelomeningocele.

## Data Availability

Data sharing is not applicable to this article.
